# B cell depletion as a therapeutic strategy for neuromyelitis optica spectrum disorder: rationale, evidence, and challenges

**DOI:** 10.3389/fimmu.2025.1635989

**Published:** 2025-08-18

**Authors:** Hirofumi Ochi, Satoru Nakamura, Jin Nakahara

**Affiliations:** ^1^ Department of Intractable Disease and Aging Science, Ehime University Graduate School of Medicine, Ehime, Japan; ^2^ Medical Affairs Department, Development and Medical Affairs Division, Mitsubishi Tanabe Pharma Corporation, Tokyo, Japan; ^3^ Department of Neurology, Keio University School of Medicine, Tokyo, Japan

**Keywords:** B cells, disease mechanisms, mechanism of action, neuromyelitis optica spectrum disorder, pathophysiology, therapeutic targets

## Abstract

Neuromyelitis optica spectrum disorder (NMOSD) is a rare autoimmune disorder of the central nervous system that predominantly affects the spinal cord and optic nerves. Aquaporin-4 antibodies have been identified as a distinguishing biomarker of NMOSD, allowing for differentiation from multiple sclerosis and other mimicking neurological conditions. Targeted monoclonal antibody treatments are evolving based on an improved understanding of the pathophysiology underlying NMOSD. Of particular influence is the idea that NMOSD is an autoantibody-mediated disease involving B cells. The hope is that targeted treatments will improve not only outcomes but also the impact and burden of the disease on patients. This review summarizes the latest evidence for B cell pathophysiology in NMOSD and highlights the cellular and molecular mechanisms of B cell-driven disease. Finally, we focus on the mechanisms of action of B cell-targeted therapies as they relate to the mechanisms of disease.

## 1 Introduction

In the late 1800s, Eugène Devic first described neuromyelitis optica (NMO), a condition characterized by acute myelitis and optic neuritis (1). Given the clinical similarities, NMO was originally thought to be a subtype of multiple sclerosis (MS), an autoimmune disease that causes inflammation in the central nervous system. In the early 2000s, Lennon et al. identified NMO-related immunoglobulin G (NMO-IgG) as a potential biomarker able to distinguish NMO from MS (2). This discovery established the concept of NMO as a separate disease. Subsequently, Lennon et al. found that NMO-IgG was a pathogenic autoantibody against the aquaporin-4 (AQP4) water channel protein (3), and NMO-IgG is now commonly referred to as AQP4 antibody (AQP4-Ab). AQP4 autoimmunity is associated with multiple clinical variants of NMO, and distinct brain lesions are seen in some cases of otherwise typical NMO. As such, these discoveries have led to the expansion of the concept of NMO to neuromyelitis optica spectrum disorder (NMOSD) (4).

The identification of AQP4-Ab as a distinguishing biomarker informed the development of diagnostic tests that differentiate NMOSD from MS and other similar neurological conditions (5). AQP4-Ab is used in the diagnosis of NMOSD (4); however, it should be noted that AQP4-Ab testing can yield both false positive and false negative results and it may be necessary to retest some patients. Furthermore, there is a subset of patients who fulfill NMOSD criteria but are AQP4-Ab seronegative (2, 4). These patients may have autoantibodies that bind to myelin oligodendrocyte glycoprotein, which is in the outer myelin sheath of neurons in the central nervous system (6, 7). They tend to have fewer disease episodes than those who are AQP4-Ab seropositive (8). Consideration must also be given to AQP4-Ab that is present in patients with disorders other than NMO and fall under the “umbrella” term of NMOSD (e.g., 30%–60% of patients with opticospinal MS are AQP4-Ab-positive) (9).

In 2015, the International Panel for NMO Diagnosis established specific diagnostic guidelines for NMOSD (4). This replaced the prior 2006 criteria, which were less specific and required the exclusion of other diagnoses. The updated guidelines incorporate both AQP4-Ab serostatus and the requirement of certain clinical features specific to NMOSD into the diagnostic criteria. They also allow for the diagnosis of AQP4-Ab seronegative NMOSD in patients with specific clinical presentations and magnetic resonance imaging findings. These criteria help distinguish NMOSD from MS by considering clinical, radiological, and serological evidence. Furthermore, McDonald’s diagnostic criteria, which are used to facilitate the diagnosis of MS, can be used to rule out MS for patients with NMOSD, ensuring they receive appropriate treatment (10, 11).

Patients with NMOSD experience attacks (i.e., relapses or flare ups) that often lead to cumulative disability including vision and sensory loss, weakness, and bladder dysfunction. The prognosis of untreated NMOSD is poor; approximately half of untreated patients will need to use a wheelchair and will lose their sight, while one-third of untreated patients die from disease-associated complications within 5 years of their first attack (12). Relapses can be severe and are associated with substantial physical, emotional, social, and financial burdens (13). Attack prevention is critical to avoiding cumulative disease-related injury (14), and for these reasons, early and effective preventative measures must be prioritized over waiting and retreating patients when their clinical symptoms re-emerge (15).

Traditionally, non-specific therapies to prevent relapse have included immunosuppression with azathioprine, tacrolimus, mycophenolate mofetil, or prednisolone (16). However, such treatments have some limitations, including the lack of assessment in randomized clinical trials (14), unsatisfactory efficacy (17), varying relapse-free rates ranging from 30%–80% (18), and high rates of adverse events and discontinuations (19).

Progress in the understanding of NMOSD has led to the development of newer, targeted monoclonal antibody treatments that improve outcomes and reduce the impact and burden of disease (20). Given the rapidly evolving treatment landscape—the first targeted agent for NMOSD was approved in 2019—there might be reservations to switching patients from the traditional treatments of oral corticosteroids and common immunosuppressive agents to the newer, highly effective disease-modifying therapies (21). A better understanding of the latest evidence supporting the use of these newer therapies might promote increased clinical use.

Given that treatment success is a moving target, it is important for clinicians managing patients with NMOSD to stay up to date on advancements in targeted B cell-depleting therapy. The rationale and clinical data supporting this approach should also be considered. The objective of this review is to summarize the latest evidence for B cell pathophysiology in NMOSD within the context of historical treatment perspectives. The cellular and molecular mechanisms of B cell-driven disease and position of B cell-targeted therapies in the context of B cell pathology are also highlighted, with a focus on mechanisms of action as they relate to the mechanisms of disease.

## 2 Pathophysiology of NMOSD

### 2.1 The role of AQP4-Ab in the pathogenesis of NMOSD

**Figure 1:**
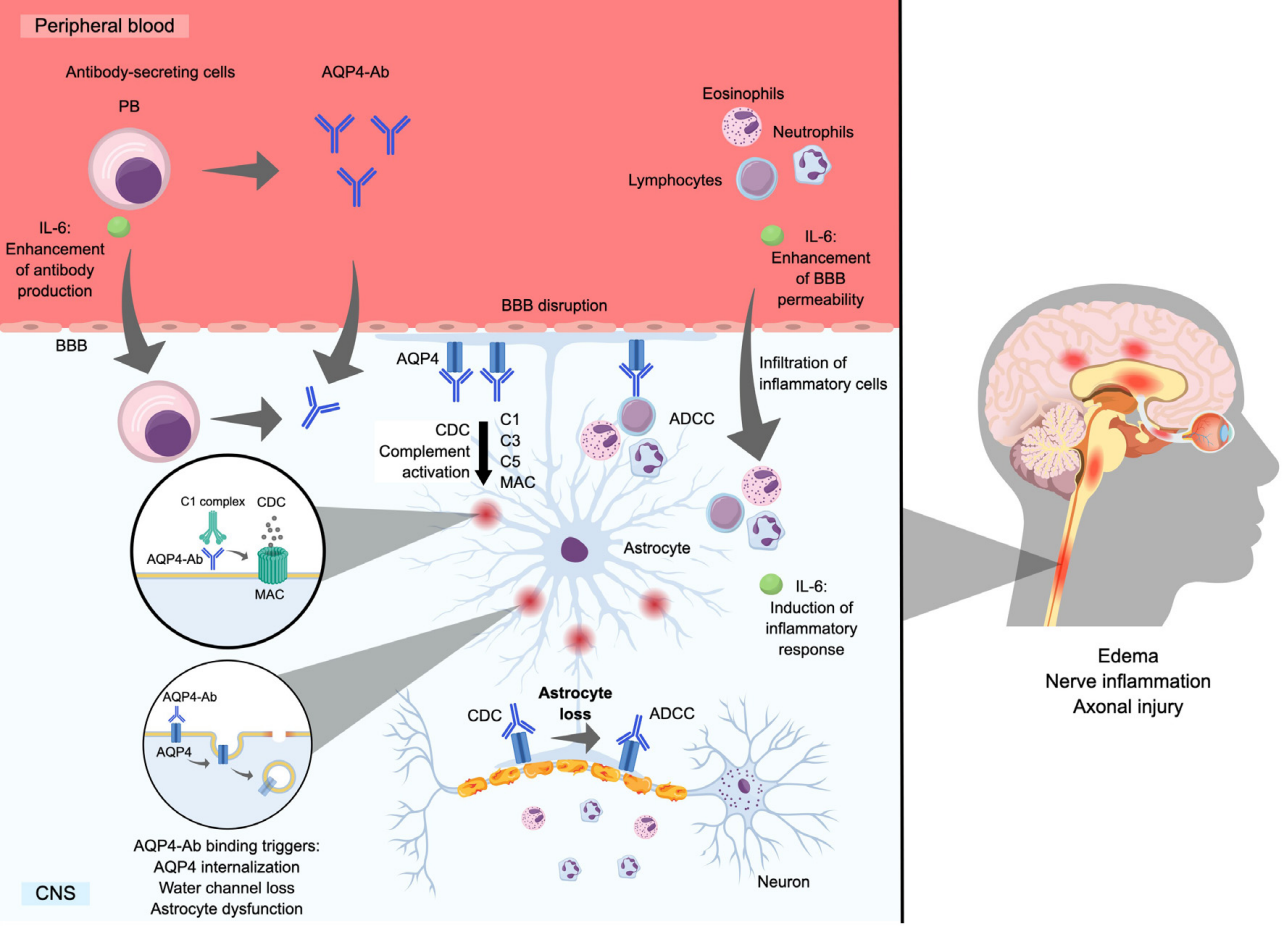
Mechanism of astrocyte loss by AQP4-Ab. Ab, antibody; ADCC, antibody-dependent cell-mediated cytotoxicity; AQP4, aquaporin-4 water channel protein; BBB, blood–brain barrier; C, complement component; CDC, complement-dependent cytotoxicity; CNS, central nervous system; IL, interleukin; MAC, membrane attack complex; PB, plasmablast; PC, plasma cell.

**Figure 1** demonstrates the pathogenic effects of AQP4-Ab in patients with NMOSD. An *in vitro* study demonstrated that exposure of astrocytes to AQP4-Ab results in a disruption of the blood–brain barrier and an increase in the production of interleukin (IL)-6 by astrocytes, resulting in enhanced leukocyte migration (22). The compromised blood–brain barrier allows AQP4-Ab in the periphery to enter the central nervous system (23) and bind to AQP4, which is highly expressed on the surface of astrocyte end-feet located near blood vessels. Antibody binding leads to complement-dependent and antibody-dependent cytotoxicity (i.e., cell death), AQP4 internalization, and disruption of water channel function (14), all of which lead to the clinical manifestations of NMOSD.

In AQP4-Ab-positive NMOSD, the main pathogenic mechanism involves complement-dependent cytotoxicity, which results in the infiltration of granulocytes and lymphocytes, causing inflammation, cell death, and tissue damage (19, 24, 25). Additionally, antibody-dependent cell-mediated cytotoxicity can occur and is primarily mediated by activated microglia, monocytes, and neutrophils (26, 27). Both mechanisms contribute to astrocyte destruction, neuroinflammation, axonal injury/loss, and demyelination in NMOSD lesions, which can become necrotic (14, 19, 25, 26, 28). The binding of AQP4-Ab to AQP4 on the surface of astrocytes is associated with the internalization of AQP4, which results in reduced glutamate uptake and impaired astrocyte water flux, leading to astrocyte dysfunction and pathological changes such as edema and inflammation (29).

Together, these mechanisms result in extensive and severe lesions that predominantly form in the optic nerves and spinal cord and lead to neurological deficits (14, 19, 25, 26, 28). These lesions are evident on magnetic resonance imaging, which shows long spinal cord lesions involving three or more vertebral bodies (8). Moreover, clear pathological features associated with the loss of AQP4 channels have been documented (8, 30, 31), and this pathology is not observed in MS (32).

Of note, it has been suggested that not all AQP4-Abs are equally pathogenic, as they are expected to have variability in their affinity and epitope specificity, which would ultimately affect their pathogenic potential (33). Indeed, evidence to date supports the notion that AQP4-Abs comprise a group of antibodies with varying binding properties against AQP4 (34), highlighting the complex pathology of NMOSD.

### 2.2 Immunotherapeutic targets based on the pathophysiology of NMOSD

Complement inhibition is an important strategy for reducing tissue damage in patients with NMOSD. It has been suggested that complement activation via AQP4-Ab drives disease activity (28). The importance of the complement system in NMOSD relapse has been demonstrated in clinical practice with the anti-C5 antibody eculizumab. In a randomized, double-blind, placebo-controlled trial, patients with AQP4-Ab-positive NMOSD who were treated with eculizumab had a significantly lower annualized relapse rate compared with those who received placebo (0.02 *vs* 0.35; rate ratio = 0.04; P <0.001) (35). Therapeutic plasma exchange has also been suggested as an important strategy for refractory acute attacks in patients with NMOSD (36) as this removes harmful humoral inflammatory mediators, including complement components, autoantibodies (i.e., AQP4-Ab), cytokines, and chemokines from the blood (37).

Given that antibodies are necessary to initiate the classical complement pathway, a reduction in the level of AQP4-Ab would be expected to lower complement activation and subsequently limit tissue damage. NMOSD disease activity is reportedly linked to levels of both peripheral AQP4-Ab and AQP4-Ab-secreting cells (i.e., plasmablasts and plasma cells), and elevated AQP4-Ab levels may be a predictor of future relapse (19). Considering their role in the pathogenesis of this disease, targeting AQP4-Ab is important for the treatment of NMOSD. Therapies associated with reduced AQP4-Ab levels prevent relapse, reduce damage to the central nervous system, restore neurological function, and improve overall outcomes in patients with NMOSD. For example, becoming AQP4-Ab seronegative following immunosuppressant treatment is associated with a lower rate of relapse compared with patients who do not become seronegative (38). Further, as we discuss in the subsequent sections, in many patients AQP4-Ab titers are reduced during B cell depletion treatment, indicating a correlation between AQP4-Ab levels, disease activity, and treatment responses (39). B cell depletion may also benefit patients via other immunomodulatory mechanisms. While largely unexplored in patients with NMOSD, these may include changes in cytokine levels, depletion of pathogenic B cell subsets, and modulation of T/B cell interactions (40–43).

Regarding treatment of NMOSD, the main therapeutic targets other than the complement cascade are IL-6 signaling and AQP4-Ab-producing cells. Interactions between autoreactive B cells and T cells in the periphery induce the production of cytokines (44), especially IL-6, which triggers the differentiation and activation of B cells into plasma cells and acts as a growth factor for antibody-secreting plasmablasts, supporting their survival and enhancing their ability to secrete AQP4-Ab (45, 46).

## 3 Mechanism of production of AQP4-Ab

### 3.1 Antibody production by plasmablasts and plasma cells

**Figure 2:**
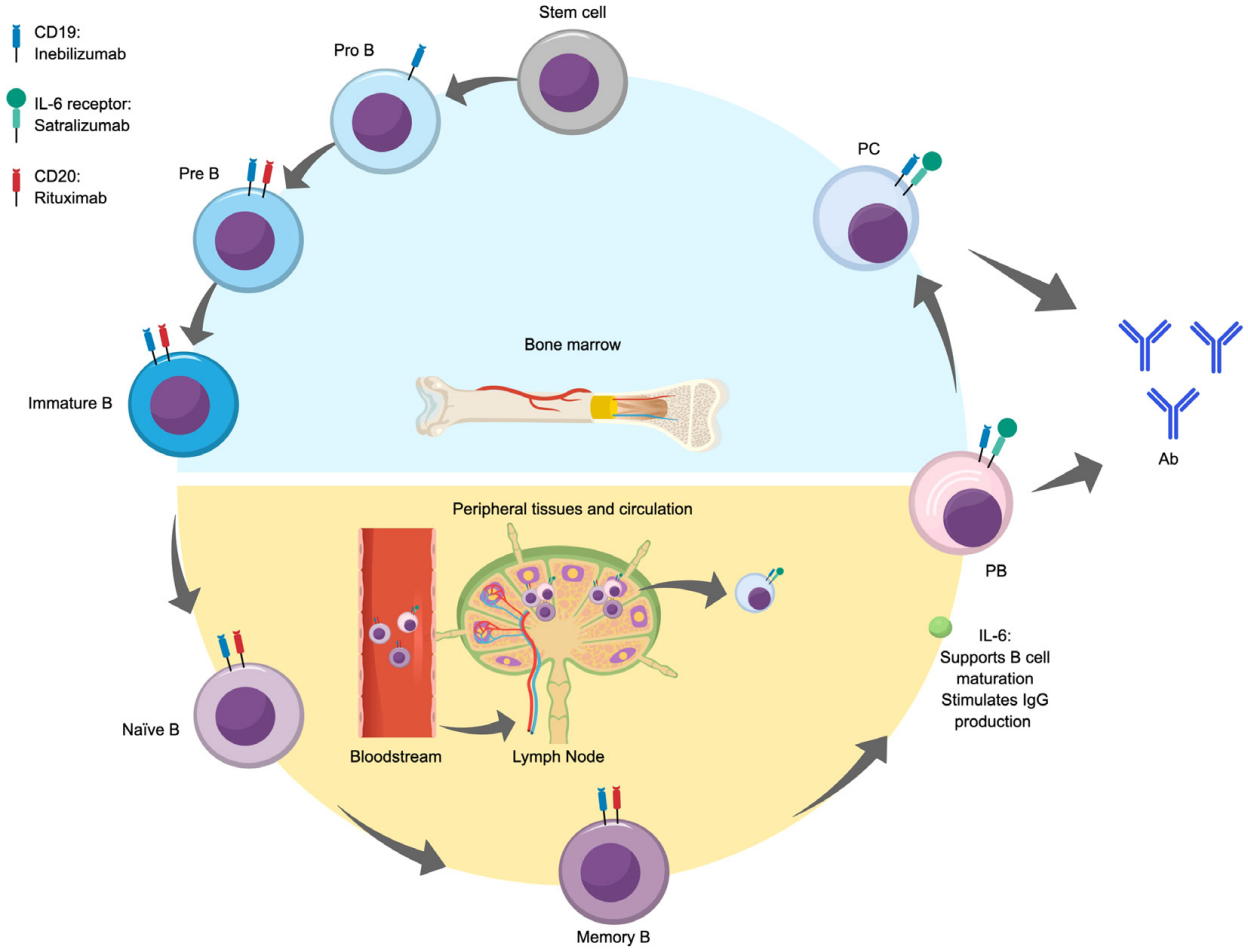
B cell differentiation and maturation and antibody production. Ab, antibody; IL, interleukin; PB, plasmablast; PC, plasma cell.

**Figure 2** illustrates the stages of differentiation and maturation of B cells from hematopoietic stem cells in the bone marrow through to antibody-producing plasmablasts and plasma cells. The differentiation and maturation of hematopoietic stem cells in the bone marrow into pro-B cells and immature B cells give rise to naïve B cells. In the early stage of differentiation, naïve B cells are exposed to an antigen, which they present to follicular effector T cells in germinal centers. Next, the cells undergo isotype switching and become memory B cells, which further differentiate into plasmablasts following re-exposure to the initial antigen via interactions with peripheral helper T cells (47, 48). IL-6 is highly involved in the life cycle of B cells, where it acts as a B cell growth factor, increases B cell survival, supports B cell maturation and plasma cell differentiation, and stimulates IgG production (49).

### 3.2 Why do autoantibodies against AQP4 develop?

B cell dysfunction is a well-recognized phenomenon in NMOSD, and specific B cell attributes have been linked to NMOSD pathology, including alterations in their number and function, the production of AQP4-specific autoantibodies by autoreactive B cells, and the production of inflammatory cytokines that can activate T cells (50). In NMOSD, B cells that escaped immune tolerance to self-antigens may differentiate into plasmablasts and plasma cells that produce AQP4-Ab (45). This immune system dysregulation occurs via three mechanisms: thymic tolerance, early B cell tolerance, and activation and differentiation of B cells. Regarding thymic tolerance, normally, thymic B cells express AQP4 in a CD40-dependent manner, which promotes the negative selection of AQP4-specific T cells, preventing their survival. However, if this mechanism fails, AQP4-specific T cells are not eliminated, and they are able to provide B cell co-stimulation in the germinal center, supporting the differentiation and survival of autoreactive B cells (51). With early B cell tolerance checkpoints, processes such as anergy induction, activation-induced cell death, or receptor editing usually prevent the formation of autoantibodies. When these checkpoints are impaired or circumvented, autoantibodies can be produced (52). Finally, during the process of B cell activation and differentiation, B cells can be activated by CD40-ligand and cytokines such as IL-21, which promote the expression of AQP4 and its presentation to T cells. This interaction is crucial for the generation of AQP4-Ab (53).

AQP4-specific autoantibodies are generated by autoreactive and polyreactive naïve B cells that are activated because of defective B cell tolerance checkpoints (14, 54). This primarily occurs in germinal centers in peripheral lymphoid tissue where T cells play a crucial role in facilitating AQP4-Ab production from plasmablasts (14, 55). AQP4-Ab can also activate T helper 17 cells to produce IL-17 (20), and both cytotoxic T cells and T helper 17 cells are involved in the pathogenesis of NMOSD. In addition, IL-6 produced by B cells can activate cytotoxic T cells, stimulate differentiation of T helper 17 cells, suppress regulatory T cell differentiation, and drive production of IL-21 from CD4^+^ T cells (17, 20).

A recent *post hoc* analysis of the N‐MOmentum study by Bennett et al. investigated the expansion of different B cell subsets during an NMOSD attack (41). The authors found no correlation between CD27^+^ memory B cells and disease activity; however, they noted that increased plasmablast and plasma cell signatures were associated with disease activity. In addition, AQP4-Ab titers were increased from baseline to the time of attack in a significant number of untreated patients, but not all. While there is currently no direct link between specific B cell subsets and AQP4-Abs with more or less pathogenic potential, the association of specific B cell subsets with disease activity, together with the emerging evidence that AQP4-Abs can vary in their pathogenic potential, make this an exciting area for further research into the pathogenic mechanisms of NMOSD.

### 3.3 Intrafollicular and extrafollicular B cell activation

**Figure 3:**
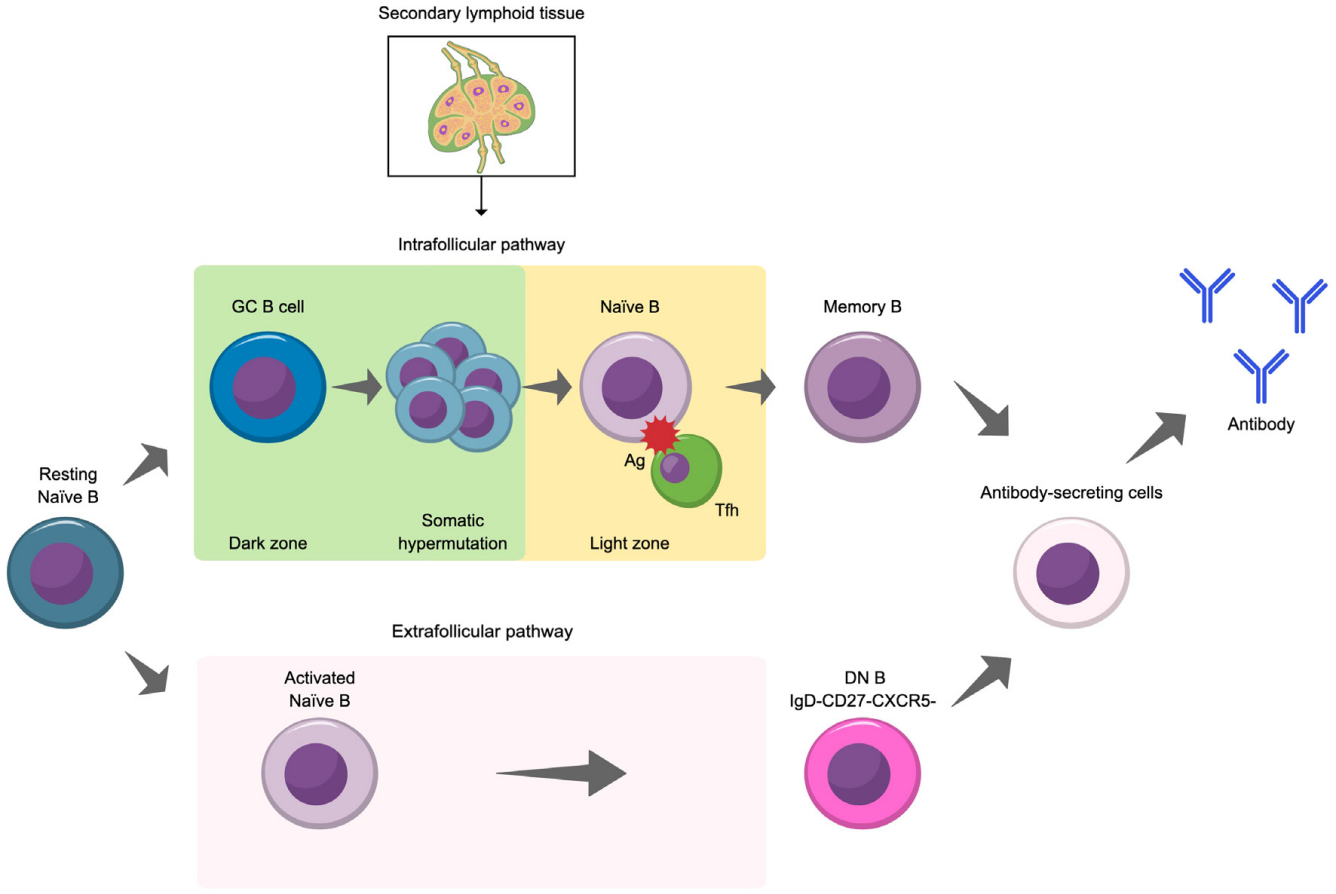
Illustration of the two pathways for the differentiation of naïve B cells into antibody secreting cells. Ag, antigen; DN B, double negative B cell; GC, germinal center; Tfh, follicular helper T cell.

As illustrated in **Figure 3**, there are two pathways in which B cells differentiate into antibody-producing cells: the intrafollicular pathway, which occurs within germinal centers, and the extrafollicular pathway, which occurs outside germinal centers (56). There are some key differences between these two pathways. Germinal center B cell activation is a relatively slow process that takes place within secondary lymphoid tissues (e.g., spleen and lymph nodes) that contain structures called follicles. During this process, germinal centers transiently form within these follicles as naïve B cells begin to interact with follicular helper T cells (57). The germinal center consists of dark and light zones (58, 59) with the dark zone mainly made up of densely packed, rapidly proliferating B cells called centroblasts, follicular T helper cells, and follicular dendritic cells. Meanwhile, the light zone is less densely packed and mainly contains B cells with higher levels of surface immunoglobulin (*vs* centroblasts) called centrocytes and follicular T cells (lower density compared with the dark zone) and follicular dendritic cells (higher density compared with the dark zone).

The interactions between B cells and follicular helper T cells facilitate the selection of high affinity B cells for differentiation into memory B cells and long-lived antibody-secreting plasma cells and support isotype switching and B cell proliferation (56). Plasma cells produce antibody upon re-exposure to their cognate antigen and with the support of helper T cells (58). Extrafollicular B cell activation is a rapid response that also plays a pivotal role in the immune response. In this pathway, which is a relatively fast process, naïve B cells interact with extrafollicular helper T cells outside of germinal centers (56). This drives B cell expansion and differentiation into short-lived plasmablasts and plasma cells. Notably, this pathway facilitates the emergence of double negative B cells (CD27^–^IgD^–^), a B cell subset that provides a robust extrafollicular response (60). While double negative B cells are a rare subset (approximately 5% of all peripheral B cells in healthy individuals), their number are expanded in several diseases, including NMOSD and other autoimmune diseases, and may contribute to their pathogenesis (60–64). As described in greater detail below, abnormalities in both pathways are observed in NMOSD.

Abnormalities in antibody production are closely related to the development of antibody-mediated autoimmune diseases. More recently, abnormalities in follicular helper T cells and CXCR4^+^ extrafollicular helper T cells have been identified as one of the factors contributing to the development of such diseases.

As noted, intrafollicular B cell activation occurs in germinal centers, and there is evidence of follicular helper T cell and follicular effector T cell involvement in NMOSD pathology. In the context of follicular effector T cells, B cells act as antigen-presenting cells to promote follicular effector T cell development and activation (28), and reciprocally, follicular effector T cells, much like follicular helper T cells, are involved in B cell differentiation and isotype switching (19). In a mouse model, an increased level of follicular helper T cells was correlated with NMOSD disease activity (65). Additionally, depletion of these cells reduced disease activity. Currently, the role of follicular helper T cells in NMOSD immunopathology is supported only by preclinical data.

Extrafollicular B cell activation has also been associated with NMOSD. A study in humans reported a correlation between frequencies of both extrafollicular helper T cells and B cells and NMOSD disease activity (66). Furthermore, double negative B cells, which are associated with extrafollicular B cell activation, are elevated in the peripheral blood of patients with NMOSD, particularly during active disease phases (62). These cells are often associated with AQP4-reactive cerebrospinal fluid B cells (62), which are crucial in the pathogenesis of NMOSD (63). Highly self-reactive, antibody-producing double negative CD11c^hi^T-bet^+^ B cells with high antigen-presenting ability have been reported in numerous autoimmune diseases (67–69). For example, a recent study reported a higher frequency of IgD^−^CD27^−^ double negative CD11c^hi^ B cells in patients with NMO compared with healthy controls and that this was related to enhanced brain atrophy and disease severity (70). Although the frequency of CD11c^hi^ B cells correlated with the frequency of peripheral helper T cells, the role of CD11c^hi^ B cells in NMO is unclear. When a previous study analyzed the immunophenotypes of patients with NMOSD and healthy controls, it was found that patients with NMOSD have a lower number of naïve B cells and a higher number of isotype-switched memory B cells and plasmablasts compared with healthy controls (71).

## 4 Efficacy and significance of B cell depletion in NMOSD treatment

### 4.1 B cell depletion and AQP4-Ab levels

Patients with NMOSD have elevated levels of CD19^+^ B cells compared with healthy controls (45), and the level of CD19^+^ B cells in patients with NMOSD correlates with the length of spinal cord lesions (72) suggesting their importance in disease. B cell dysfunction is a well-recognized phenomenon in NMOSD, and B cell depletion has shown clinical benefit, further providing a causal link between B cell dysfunction and NMOSD (73). Specific B cell attributes that are dysfunctional and linked to NMOSD pathology include B cell-produced antibodies, altered lymphocyte functions and numbers, B cell-produced cytokines, and B and T cell interactions (50).

There are several methods for inducing B cell depletion, which may influence its effect on antibody production. For example, using anti-CD20 therapy is only expected to deplete pre-B cells through some plasmablasts, while anti-CD19 therapy is expected to deplete B cells of all stages (pro-B cells through to plasma cells) (74). Because both plasmablasts and plasma cells secrete antibodies, the method of B cell depletion may affect the efficacy of these treatments in the context of AQP4-Ab-mediated pathologies of NMOSD.

Studies with rituximab (an anti-CD20 monoclonal antibody) in patients with NMOSD have reported a reduced or maintained level of AQP4-Ab following treatment (39, 75–77) and delayed treatment has been reported to be associated with an increase in AQP4-Ab levels (75). Additionally, it has been reported that relapses are often—but not always—associated with increased AQP4-Ab levels (39, 75). An exploratory analysis of the N-MOmentum study reported a significant depletion of CD20^+^ B cells in patients with NMOSD who were treated with inebilizumab (an anti-CD19 monoclonal antibody) compared with those treated with placebo after 28 weeks of treatment (78). With B cell depletion, there was a significant decrease in the plasma cell signature (indicating fewer plasma cells were present) and in total immunoglobulin levels. However, the level of IgG antibodies was unchanged with treatment; significantly decreased levels were limited to IgA, IgM, and IgE isotypes.

The *post hoc* analysis of the N-MOmentum study reported that, among patients who were AQP4-Ab seropositive at baseline, 37% treated with inebilizumab and 18% treated with placebo experienced a ≥2-fold decrease in AQP4-Ab titer from baseline (P = 0.014) (41). Among patients with the highest AQP4-Ab titers at baseline (≥1:20,480), 51% and 8% of patients treated with inebilizumab and placebo, respectively, had a ≥2-fold titer decrease (P <0.05). This study also demonstrated that higher AQP4-Ab titers correlated with an increase in attack rate; however, the attack rate decreased over time following treatment, even among patients with the highest AQP4-Ab titers at baseline. The correlation between AQP4-Ab titers and clinical outcomes is inconsistent. For example, one study reported a correlation between higher antibody titers and increased disease severity (79) but it has also been reported that AQP4-Ab titers do not predict clinical outcomes such as relapses, relapse severity, or disability (80). Additionally, patients with NMOSD who are AQP4-Ab seronegative still experience attacks, further suggesting other mechanisms of disease pathophysiology (81).

The observation that B cell depletion does not always result in reduced AQP4-Ab titers and that AQP4-Ab titers do not consistently correlate with clinical outcomes suggests a broader therapeutic impact of B cell depletion (41). This may involve the reduction of pathogenic B cell subsets and the modulation of immune interactions by potentially reducing both cytokine secretion by B cells (e.g., IL-6) and B and T cell interactions. Thus, other immunological mechanisms may contribute to the success of B cell depletion therapy for patients with NMOSD.

### 4.2 Ab-independent B cell pathophysiology of NMOSD

In patients with NMOSD, the expression of B cell-affecting cytokines (IL-6, IL-17, B cell-activating factor of the tumor necrosis factor family [BAFF], and a proliferation-inducing ligand) is elevated in the serum and cerebrospinal fluid (20). Thus, inhibition of these cytokines may be beneficial to patients. For example, the pro-inflammatory cytokine IL-6 plays a crucial role in B cell differentiation and maturation. Satralizumab, a humanized monoclonal antibody that targets the IL-6 receptor, has shown clinical efficacy by significantly reducing the annualized relapse rate in NMOSD clinical trials compared with placebo-treated patients (82, 83).

B cells play a role in antibody-independent pathologies of NMOSD, for example, B cells release cytokines that activate the innate immune system, contributing to disease progression (84). A subset of B cells, called regulatory B cells, function to suppress immune responses (85). These cells produce anti-inflammatory cytokines, such as IL-10, IL-35, and transforming growth factor-β, and play an important role in maintaining immune homeostasis. However, the regulatory function of B cells is attenuated in patients with NMOSD, contributing to the disease pathology (28).

Although NMOSD primarily involves B cells, it has been suggested that T cells may also indirectly contribute to the pathophysiology of NMOSD as they are essential for AQP4-Ab production from plasmablasts (14). B cells are thought to act as antigen-presenting cells for AQP4, priming autoreactive T cells and stimulating their differentiation into T helper 17 cells (86). In turn, T helper 17 cells support B cells in becoming AQP4-Ab-producing plasma cells.

### 4.3 Tailoring B cell depletion for NMOSD

**Figure 4:**
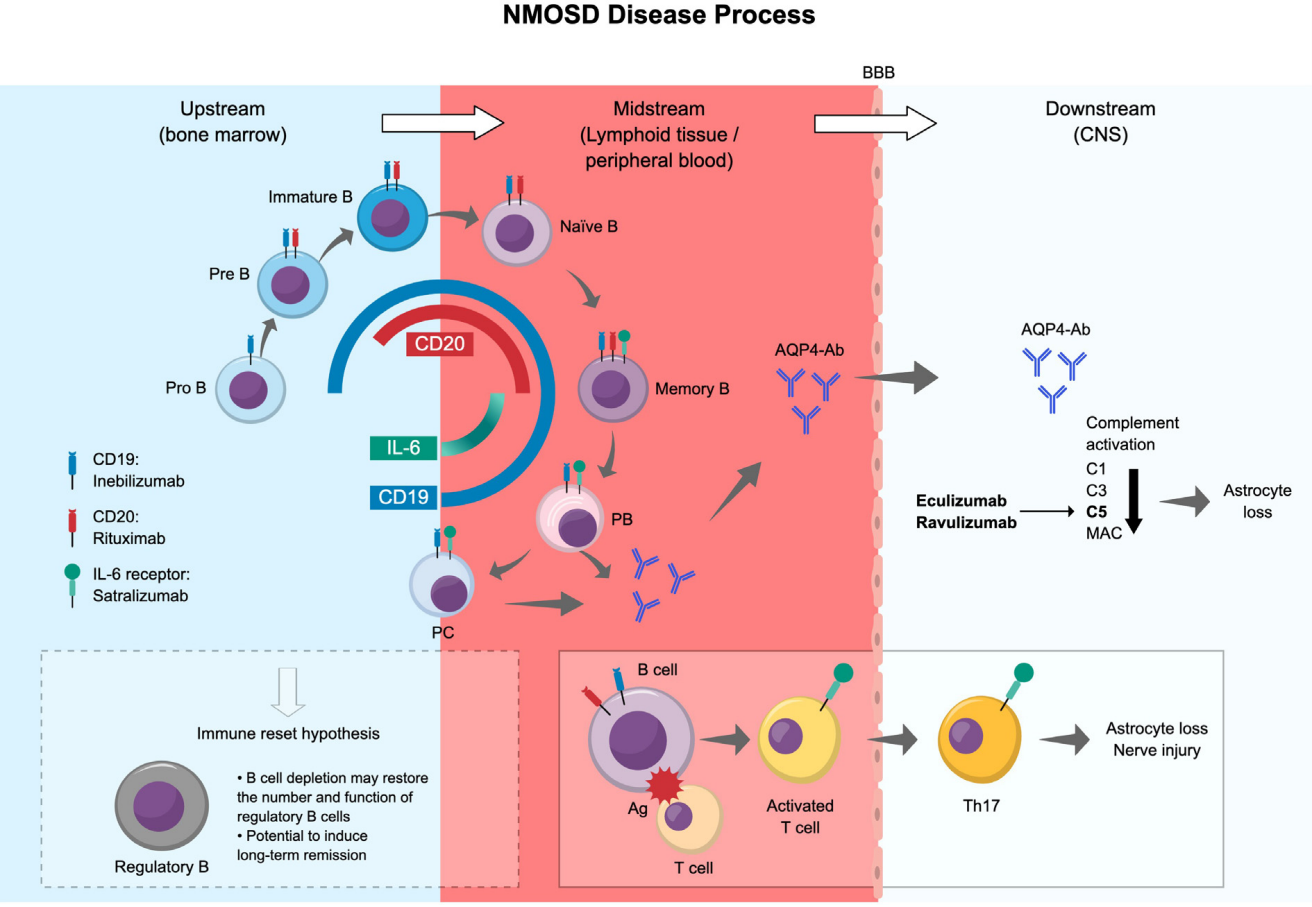
Summary of the NMOSD disease process and differences in the effects of antibody products. Ag, antigen; AQP4-Ab, aquaporin-4 water channel protein-specific antibody; BBB, blood–brain barrier; C, complement component; CNS, central nervous system; DN B, double negative B cell; IL, interleukin; MAC, membrane attack complex; NMOSD, neuromyelitis optica spectrum disorder; PB, plasmablast; PC, plasma cell.

Understanding that B cells play a pivotal role in the pathogenesis of NMOSD through the production of pathogenic AQP4-Ab and other non-antibody-mediated pathways has led to new, promising therapeutic strategies for NMOSD, particularly through targeted B cell depletion. Of note, there has been no head-to-head study of the effects of CD19- and CD20-depleting treatments for NMOSD. **Figure 4** summarizes the NMOSD disease process and the differences in the effects of monoclonal antibody products targeting B cells, IL-6, and the C5 complement component.

The expression and distribution of cell surface markers such as CD19 and CD20 are important for identifying and selectively targeting B cells (87). Anti-CD19 has an advantage over anti-CD20 in that it can eliminate a broader range of B lineage cells, including pathogenic AQP4-Ab-producing plasma cells and juvenile early pro-B cells. It is noteworthy that even a small emergence of CD27^+^ memory B cells increases the risk of recurrence (75). While a correlation has been reported between deep and persistent CD20^+^ B cell depletion and long-term clinical stability, early, deep B cell depletion has been found to correlate with improved disease activity (78, 88). CD19-expressing high antibody-producing B cells (CD27^–^IgD^–^ double negative CD11c^hi^T-bet^+^), which we introduced earlier in this review, might be depleted by anti-CD19 treatment. However, plasmablasts and double negative B cells, which are involved in antibody production and increased in the peripheral blood of patients with NMOSD, express low levels of CD20 (62, 78), and therefore may be difficult to deplete deeply with CD20 antibodies.

Several clinical trials evaluating B cell counts following treatment with CD19- (inebilizumab) and CD20- (rituximab) targeting therapies in patients with NMOSD have been conducted to date, providing insights into the effectiveness of each targeted therapy. The N-MOmentum study showed that inebilizumab treatment of patients with NMOSD rapidly eliminated circulating total CD20^+^ B cells and lowered annual attack rates (78). Long-term results of the N-MOmentum study showed that inebilizumab treatment induced a robust depletion of B cells that was maintained over 4 years and stabilized disability scores (89). Furthermore, a *post hoc* analysis of the N-MOmentum study reported that patients with NMOSD who had breakthrough attacks, despite previous treatment with rituximab, derived benefit from treatment with inebilizumab related to reduced breakthrough attacks (90). However, participants were more susceptible to infection.

Because CD20 is not expressed by pathogenic AQP4-Ab-producing plasma cells, rituximab likely mediates its effects via non-antibody related mechanisms. A long-term study evaluating the effect of CD20-targeted therapy on B cell counts in patients with NMOSD showed that rituximab depleted B cells from the blood and cerebrospinal fluid, but its effect on AQP4 antibody titers was unclear (91). Regarding a potential non-antibody related mechanism of action of rituximab, a recent study showed that in patients with NMOSD, rituximab treatment led to restored numbers and functions of replenished regulatory B cells (92). These CD24^hi^CD38^hi^ B cells regained the ability to produce IL-10, which suppressed pro-inflammatory cytokine (interferon-γ, IL-17) production from CD4^+^ T cells. This so-called “immune reset hypothesis” posits that B cell depletion might lead to the reconstitution of regulatory B cells with the potential to induce long-term remission in patients with NMOSD. However, further studies will be needed to confirm this phenomenon for B cell-targeted therapies such as rituximab and inebilizumab. Another study reported that treatment with rituximab reduced Th17 cell responses in patients with rheumatoid arthritis (93). This may be relevant for patients with NMOSD, as a shift towards a T helper 1/T helper 17 and T helper 17/Treg pro-inflammatory immune response is associated with disease activity and severity (94). Further studies are needed to identify the effects of B cell depletion beyond AQP4-Ab reduction in patients with NMOSD.

Correlations between B cell counts and NMOSD disease activity have been reported in both inebilizumab- and rituximab-treated patients (78, 91), which is important as monitoring B cell counts may help guide treatment decisions, predict relapse, and assess the effectiveness of treatment. Overall, this would allow clinicians to provide more targeted care for patients with NMOSD.

### 4.4 Clinical concerns and counterpoints for using B cell-targeting agents

Concerns exist about an increased theoretical risk of serious infections (including COVID-19) with long-term use of B cell-depleting agents (14, 17). However, in a 14-year follow-up study of rituximab in patients with NMOSD, infection rates remained low and did not correlate with IgG levels despite the reduced levels (95). Additionally, there are concerns that the humoral response following vaccination for COVID-19 was impaired in patients with NMOSD treated with B cell-depleting agents (17, 96). Nevertheless, while B cells in the blood may be depleted, remaining tissue-resident B cells may provide some immune protection, alongside T cell-mediated immunity (97). Furthermore, some antibody production is sustained by CD19^−^ long-lived plasma cells in the bone marrow, even after B cell removal (98, 99). Further research is warranted to address concerns about the long-term safety impact of B cell removal and IgG reduction (21).

Differences in the expression of CD19 and CD20 on late-stage B cells (such as plasmablasts and plasma cells) affect the outcome of B cell targeted therapies. The choice between targeting CD19 or CD20 for B cell depletion therapy must consider the optimum balance between effectiveness and safety. Given that CD19 is more widely expressed on B cell lineages than CD20, CD19-targeted therapies result in broader B cell elimination than those targeting CD20. However, this longer-lasting effect could lead to a higher risk of immunosuppression. CD20-targeted therapies such as rituximab do not target antibody-secreting cells such as plasma cells, so the production of AQP4-Ab might persist, leading to the need for ongoing or repeated treatments. While more aggressive B cell depletion with CD19-targeted therapy may be preferred in patients with higher risks of severe disease or frequent relapse, CD20-targeted therapy may be the preferred choice for patients with more stable disease or those with a higher risk of infection. However, CD20 is expressed by other non-B cell immune cells such as T cells and neutrophils, which might lead to adverse events such as neutropenia and T cell hyporesponsiveness.

After B cell depletion, the appearance of BAFF may lead to a resurgence in pro-inflammatory B cells (40). However, a recent publication on MS and a model of experimental autoimmune encephalomyelitis suggests that BAFF is neuron-protective, and thus could also have a beneficial effect (100).

Genetic factors, such as the presence of Fc gamma receptor IIIa (*FCGR3A*) gene polymorphisms, are also important for tailoring B cell depletion therapy for patients with NMOSD, as they are known to affect the cytotoxicity of monoclonal antibody drugs. The F allele polymorphism at amino acid 158 *FCGR3A* polymorphism has been shown to negatively affect the efficacy of rituximab (CD20-targeting therapy) but not inebilizumab (CD19-targeting therapy) (101). Therefore, genetic testing may be useful in the treatment decision-making process to help optimize outcomes and minimize the risk of ineffective treatment.

## 5 Future perspectives and conclusions on B cell-removal therapies as a therapeutic strategy for NMOSD

In the N-MOmentum study evaluating inebilizumab, a few incidences of relapse were observed in the early stages of inebilizumab treatment, but after approximately 1 year of treatment no relapse events were recorded (78, 88, 89). One potential reason for this may be the study design, which required the concomitant use of steroids to be completely discontinued at the start of inebilizumab treatment. The depth and extent of B cell depletion over time may also account for this, although further studies are necessary to clarify this (89). Another possible explanation is that an immune reset, which may involve the restoration of regulatory B cells, may take some time to develop and thus the effects may not have been observed early in the study.

Immune reset is the reconstitution of the immune system after B cell depletion; that is, the removal of pathogenic B cells and the regeneration of normal B cells are postulated to restore the immune balance. It has been suggested that B cell depletion therapy with rituximab may modulate the pathogenesis of NMOSD by inducing the reconstitution of regulatory B cells such as CD 19^+^CD24^hi^CD38^hi^ B cells (92). This reconstitution may suppress relapses in the long term, potentially freeing patients from ongoing drug treatment regimens. However, a murine model of MS showed that, after CD20-targeted therapy, there was an elevated frequency of myelin-reactive B cells, suggesting that pathogenic B cells may persist or reappear after B cell depletion (102). It has also been reported that 92.5% of patients seropositive for AQP4-Ab had reemergence of memory B cells after rituximab treatment (103). These observations may imply a need for retreatment, which is supported by a report stating that relapses may occur with delayed rituximab retreatment in patients with NMOSD (104). Furthermore, assessing immune reset involves more than analyzing circulating lymphocytes, a thorough examination of B cell repertoires in organs and tissues beyond the blood is required. The effects on regulatory B cells and recovery have not yet been confirmed with CD19-targeted therapies such as inebilizumab. Further studies are therefore needed to verify the immune reset phenomenon following B cell depletion.

A recent report linking circulating CD11c^+^ B cells to brain atrophy in patients with NMOSD emphasizes the need to study whether B cell-depleting therapies can prevent relapse and progression of brain lesions in NMOSD patients over time (70). The concept that NMOSD progresses mainly through relapses rather than continuous asymptomatic changes, will influence future treatment approaches, emphasizing the need to focus on preventing both symptomatic relapses and subclinical disease activity. Additionally, there is strong interest in using biomarkers to determine disease status and response to treatment in future treatment decisions for NMOSD (105). For B cell-depleting therapies using rituximab or inebilizumab, blood B cell counts during treatment may be a useful biomarker to determine the duration of drug response.

Future research should focus on understanding the impact of switching from other biologics in NMOSD and determining whether the reconstitution of B cells occurs after inebilizumab treatment as it does with rituximab treatment (92). Furthermore, efforts to understand the contribution of AQP4-Ab titers and antibody-independent mechanisms to the pathogenesis of NMOSD should be continued, as such studies will potentially inform improved treatment strategies. Overall, the primary objective in NMOSD treatment is to prevent relapse, and therefore it is critically important to use biologics with the correct mechanism of action that aligns with a patient’s specific background characteristics.
